# Correction: Functionalized graphene-based materials for cementitious applications

**DOI:** 10.1039/d4ra90008a

**Published:** 2024-02-05

**Authors:** Andrea Cacciatore, Paolo Zardi, Claudia Capone, Michele Maggini

**Affiliations:** a Italcementi S.p.A.-Heidelberg Materials Via Stezzano, 87 24126 Bergamo Italy; b Dipartimento di Scienze Chimiche, Università di Padova Via F. Marzolo 1 35131 Padova Italy; c Istituto di Chimica della Materia Condensata e di Tecnologie per l'Energia – CNR Corso Stati Uniti 4 35127 Padova Italy michele.maggini@unipd.it

## Abstract

Correction for ‘Functionalized graphene-based materials for cementitious applications’ by Andrea Cacciatore *et al.*, *RSC Adv.*, 2024, **14**, 3314–3320, https://doi.org/10.1039/D3RA06886B.

The authors regret that the name of one of the authors (Claudia Capone) was shown incorrectly in the original article. The corrected author list is as shown above.

The authors regret the omission of an acknowledgement in the original article. This acknowledgement is given below.

We thank S. Pressi and E. Menna for their assistance in the GBM functionalization and characterization.

The Royal Society of Chemistry apologises for these errors and any consequent inconvenience to authors and readers.

## Supplementary Material

